# African American patients with gout: efficacy and safety of febuxostat vs allopurinol

**DOI:** 10.1186/1471-2474-13-15

**Published:** 2012-02-09

**Authors:** Alvin F Wells, Patricia A MacDonald, Solomon Chefo, Robert L Jackson

**Affiliations:** 1 Rheumatology and Immunotherapy Center, 4225 W. Oakwood Park Court; Franklin, WI 53132, USA; 2 Takeda Global Research and Development Center, Inc, One Takeda Parkway, Deerfield, IL 60015, USA

## Abstract

**Background:**

African Americans are twice as likely as Caucasians to develop gout, but they are less likely to be treated with urate-lowering therapy (ULT). Furthermore, African Americans typically present with more comorbidities associated with gout, such as hypertension, obesity, and renal impairment. We determined the efficacy and safety of ULT with febuxostat or allopurinol in African American subjects with gout and associated comorbidities and in comparison to Caucasian gout subjects.

**Methods:**

This is a secondary analysis of the 6-month Phase 3 CONFIRMS trial. Eligible gouty subjects with baseline serum urate (sUA) ≥ 8.0 mg/dL were randomized 1:1:1 to receive febuxostat 40 mg, febuxostat 80 mg, or allopurinol (300 mg or 200 mg depending on renal function) daily. All subjects received gout flare prophylaxis. Primary efficacy endpoint was the proportion of subjects in each treatment group with sUA < 6.0 mg/dL at the final visit. Additional endpoints included the proportion of subjects with mild or with moderate renal impairment who achieved a target sUA < 6.0 mg/dL at final visit. Adverse events (AEs) were recorded throughout the study.

**Results:**

Of the 2,269 subjects enrolled, 10.0% were African American and 82.1% were Caucasian. African American subjects were mostly male (89.5%), obese (BMI ≥ 30 kg/m^2^; 67.1%), with mean baseline sUA of 9.8 mg/dL and mean duration of gout of 10.4 years. The proportions of African American subjects with a baseline history of diabetes, renal impairment, or cardiovascular disease were significantly higher compared to Caucasians (*p *< 0.001). ULT with febuxostat 80 mg was superior to both febuxostat 40 mg (*p *< 0.001) and allopurinol (*p *= 0.004). Febuxostat 40 mg was comparable in efficacy to allopurinol. Significantly more African American subjects with mild or moderate renal impairment achieved sUA < 6.0 mg/dL in the febuxostat 80 group than in either the febuxostat 40 mg or allopurinol group (*p *< 0.05). Efficacy rates in all treatment groups regardless of renal function were comparable between African American and Caucasian subjects, as were AE rates.

**Conclusions:**

In African American subjects with significant comorbidities, febuxostat 80 mg is significantly more efficacious than either febuxostat 40 mg or allopurinol 200/300 mg. Febuxostat was well tolerated in this African American population.

Please see related article: http://www.biomedcentral.com/1741-7015/10/15

## Background

The rate of gout in the United States has been on the rise and increases with age, in both men and women [1-3]. African Americans make up roughly 13% of the US population [4], and are twice as likely as Caucasians to develop gout [5,6]. In a prospective cohort study, 571 Caucasian and 352 African American young men were followed for a median duration of 29 years [5]. The cumulative incidence of gout in these 2 cohorts was 5.8% and 10.9%, respectively. Yet, African Americans represent only 10% of the patients treated for gout [7]. Moreover, when they are treated, they are less likely to receive urate-lowering therapy (ULT) [7]. Furthermore, African Americans typically present with higher rates of comorbidities, such as hypertension, obesity, and renal impairment [8], and have higher rates of diuretic use compared to Caucasians [9], all of which have been independently associated with hyperuricemia and gout [10].

Gout is characterized by hyperuricemia (serum urate concentration [sUA] exceeding 6.8 mg/dL, the limit of urate solubility) and acute and chronic consequences of monosodium urate crystal deposition, such as tophi and gout flares [11]. Long-term management of chronic gout with ULT focuses on achieving and maintaining sUA in a sub-saturating range (< 6.0 mg/dL) with the goal of dissolving monosodium urate crystals and decreasing the body pool of uric acid [12,13]. ULT for up to 5 years leads to the elimination of acute flares and reduction in the size and number of tophi present [14,15].

Available ULT options in the US include xanthine oxidase (XO) inhibitors allopurinol and febuxostat, and probenecid, a uricosuric. Allopurinol, which is the mainstay of chronic gout management [16], requires dose adjustments in gout patients with renal impairment [17], which may lead to reduced efficacy [18]. Febuxostat is a selective, non-purine analog XO inhibitor [19] for the treatment of chronic hyperuricemia in patients with gout [20]. Data from 3 comparative, blinded, randomized controlled trials (RCTs) have demonstrated the superior efficacy of febuxostat 80 mg daily compared with both the commonly prescribed dose of allopurinol (300 mg) [17] and placebo [21-23]. In addition, both approved doses of febuxostat, 80 mg and 40 mg, are significantly more efficacious than allopurinol (*p *< 0.001 and *p *= 0.012, respectively) in achieving the therapeutic target sUA in subjects with mild-to-moderate renal impairment [23].

There are no data from prospective RCTs specifically exploring ULT efficacy and safety in African Americans with gout. The objective of this post hoc subanalysis of the CONFIRMS trial [23], the largest ULT RCT to date, was to examine the urate-lowering efficacy and safety of febuxostat and allopurinol in hyperuricemic African American subjects with gout in comparison to Caucasian subjects.

## Methods

The 6-month CONFIRMS (NCT# 00430248) trial enrolled male and female subjects 18 to 85 years of age with a diagnosis of gout (defined by American Rheumatology Association preliminary criteria [24]) and hyperuricemia (sUA ≥ 8.0 mg/dL). Approval was obtained from Quorum Review Institutional Review Board, Seattle, WA. Subjects provided written, informed consent and Health Insurance Portability and Accountability Act authorization prior to any study-related procedure. This study was conducted with respect for the individual participating subjects according to the Declaration of Helsinki, the ICH Harmonised Tripartite Guideline for GCP, and all applicable local regulations. Exclusion criteria included secondary hyperuricemia, xanthinuria, severe renal impairment (estimated creatinine clearance [eCLcr] < 30 mL/min, calculated by Cockcroft-Gault formula corrected for ideal body weight [25,26]), alanine aminotransferase and aspartate aminotransferase values > 1.5 times the upper limit of normal, consumption of > 14 alcoholic drinks per week or a history of alcoholism or drug abuse within 5 years, or medical condition that would interfere with treatment, safety, or adherence to the study protocol. In addition, subjects with known hypersensitivity to febuxostat, allopurinol, naproxen, any other non-steroidal anti-inflammatory agents, aspirin, lansoprazole, colchicine, or any components of these drugs formulations were excluded.

Subjects were randomized 1:1:1 to receive a daily dose of either febuxostat 40 mg, febuxostat 80 mg (Uloric^®^; Takeda Global Research & Development Center, Inc; Deerfield, IL), or allopurinol (Apotex; Weston, FL, USA). Subjects randomized to allopurinol were to receive 300 mg daily if baseline renal function was normal (eCLcr ≥ 90 mL/min) or mildly impaired (eCLcr 60 to < 90 mL/min); subjects with moderate renal impairment (eCLcr 30 to < 60 mL/min) were to receive 200 mg daily.

Throughout the 6-month treatment period, subjects received prophylaxis for gout flares, self-administering either colchicine (Westward Pharmaceutical Corporation; Eatontown, NJ) 0.6 mg daily, or naproxen (Westward Pharmaceutical Corporation) 250 mg twice daily. Subjects with eCLcr < 50 mL/min were not to receive naproxen. All subjects receiving naproxen prophylaxis also received lansoprazole 15 mg daily (Takeda Global Research & Development Center, Inc).

The primary efficacy endpoint of CONFIRMS was the proportion of subjects in each treatment group who achieved a target sUA < 6.0 mg/dL at final visit [23]. In the primary CONFIRMS analysis of the total cohort (N = 2,269), non-inferiority of febuxostat 40 mg compared to allopurinol 300/200 mg dose was demonstrated. Binomial 95% confidence intervals (CIs) were calculated for the difference between the 2 groups in achieving the primary efficacy endpoint. Non-inferiority was declared if the lower limit of the 95% CI for difference in the proportion of subjects achieving an sUA < 6.0 mg/dL at the last visit was greater than -10%. The difference between the febuxostat 40 mg group (45.2%) and the allopurinol group (42.1%) was 3.1% (95% CI: -1.9% - 8.1%), thus demonstrating non-inferiority [23]. Efficacy comparisons between treatment groups were made using Fisher's exact test (two-tailed, 0.05 significance level. Additional efficacy endpoints were the proportion of subjects in each treatment group with mild and with moderate renal impairment who achieved a target sUA < 6.0 mg/dL at final visit. Pairwise comparisons were made between treatment groups with Fisher's exact test. The objectives of our current analyses were to assess the primary and additional efficacy endpoints and safety among African American subjects and compare these results with those observed in the subgroup of Caucasian subjects enrolled in the CONFIRMS trial.

All treatment-emergent adverse events (AEs) were recorded using Medical Dictionary for Regulatory Activities (MedDRA). Gout flares were not considered AEs and were reported separately.

Statistical analyses of the achievement of the efficacy endpoints for each treatment group have been previously described in detail [23]. In addition, to determine statistically significant differences between African American and Caucasian subjects for baseline characteristics of age, body mass index (BMI), baseline sUA, and years with gout, analysis of variance was used; for all other categorical baseline variables, Fisher's exact test was used. In addition, Fisher's exact test was used to determine statistical significant differences between the proportions of African American and Caucasian subjects within each treatment group who achieved the primary and additional efficacy endpoints, and differences in rates of AEs between African American and Caucasian subjects.

## Results

**Figure 1:**
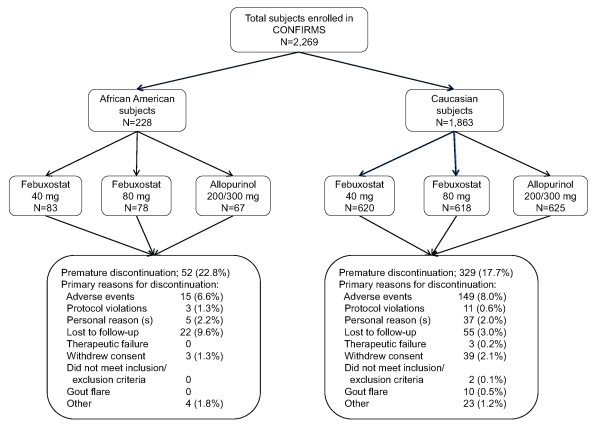
**Flow of African American and Caucasian Subjects Through the CONFIRMS Trial**.

Of the 2,269 subjects who enrolled in the CONFIRMS trial, 228 (10.0%) were African American and 1,863 (82.1%) were Caucasian. Fifty-two (22.8%) and 329 (17.7%) African Americans and Caucasian subjects, respectively, discontinued from the study prematurely (Figure 1). The most common primary reason for premature discontinuation among African American subjects was lost to follow-up (n = 22; 9.6%), while AEs were the most frequent primary reason for Caucasians (n = 149; 8.0%). Compliance with therapy (≥ 90% compliant), calculated as the number of capsules taken divided by the number of days on drug, was lower among African Americans compared to Caucasians; 72.4% vs 82.1%, respectively. When analyzed by treatment group, compliance (≥ 90%) for African American subjects in the febuxostat 40 mg, febuxostat 80 mg, and allopurinol treatment groups was 66.3%, 76.9%, and 74.6%, respectively, while it was 82.4%, 82.2%, and 81.6%, respectively, for Caucasian subjects.

At baseline, compared to the Caucasian subgroup, the African American subgroup had significantly more women (10.5% vs 5.1%; *p *< 0.001), higher mean BMI (34.0 kg/m^2 ^vs 32.8 kg/m^2^; *p *= 0.006), higher mean sUA (9.8 mg/dL vs 9.5 mg/dL; *p *< 0.001), and lower mean duration of gout (10.4 years vs 11.8 years; *p *= 0.030). In addition, the proportions of subjects with a history of diabetes, renal impairment, or cardiovascular disease at baseline were significantly higher among African Americans compared to Caucasians (*p *< 0.001; Table 1).

**Table 1:** Comparison of Baseline Demographics and Characteristics Between African American and Caucasian Subjects in the CONFIRMS Trial

Variable	African American N = 228	Caucasian N = 1,863	*p *value^a^
**Gender**, *n (%)*			
Male	204 (89.5)	1,768 (94.9)	< 0.001
Female	24 (10.5)	95 (5.1)	
**Age (years)**			
Mean ± SD	53.2 ± 10.45	53.0 ± 11.80	0.799
**Body Mass Index (kg/m^2^)**			
Mean ± SD	34.0 ± 7.47	32.8 ± 6.09	0.006
**Alcohol Use**, *n (%)*			
Non-/Ex-drinker	88 (38.6)	563 (30.2)	0.010
Drinker (1-14 drinks/week)	140 (61.4)	1,300 (69.8)	
**Tobacco Use**, *n (%)*			
Non-/Ex-tobacco User	177 (77.6)	1,528 (82.0)	0.107
Tobacco User	51 (22.4)	335 (18.0)	
**Serum Urate (mg/dL)**			
Mean ± SD	9.8 ± 1.24	9.5 ± 1.16	< 0.001
**Years with Gout**			
Mean ± SD	10.4 ± 8.28	11.8 ± 9.49	0.030
**Tophi Present**, *n (%)*			
No	169 (74.1)	1,482 (79.5)	0.058
Yes	59 (25.9)	381 (20.5)	
**Renal Function,^b ^***n (%)*			
Moderately Impaired	69 (30.3)	299 (16.0)	< 0.001
Mildly Impaired	109 (47.8)	884 (47.5)	
Normal	50 (21.9)	680 (36.5)	
**Medical History**, *n (%)*			
Any cardiovascular disease	170 (74.6)	1,024 (55.0)	< 0.001
Hypertension	165 (72.4)	937 (50.3)	< 0.001
Coronary artery disease	25 (11.0)	161 (8.6)	0.245
Cardiac arrhythmia	19 (8.3)	202 (10.8)	0.245
Diabetes	57 (25.0)	229 (12.3)	< 0.001
Hypercholesterolemia	19 (8.3)	130 (7.0)	0.454
Hyperlipidemia	83 (36.4)	776 (41.7)	0.128
Use of low-dose aspirin (≤ 325 mg daily)	40 (17.5)	343 (18.4)	0.749

^a^*p *values determined from analysis of variance model for age, body mass index, baseline serum urate, and years with gout, and by Fisher's exact test for all other variables.

^b^Moderate baseline renal impairment: estimated creatinine clearance (eCLcr) 30 to < 60 mL/min; mild baseline renal impairment: eCLcr 60 to < 90 mL/min; normal: eCLcr ≥ 90 mL/min.

**Figure 2:**
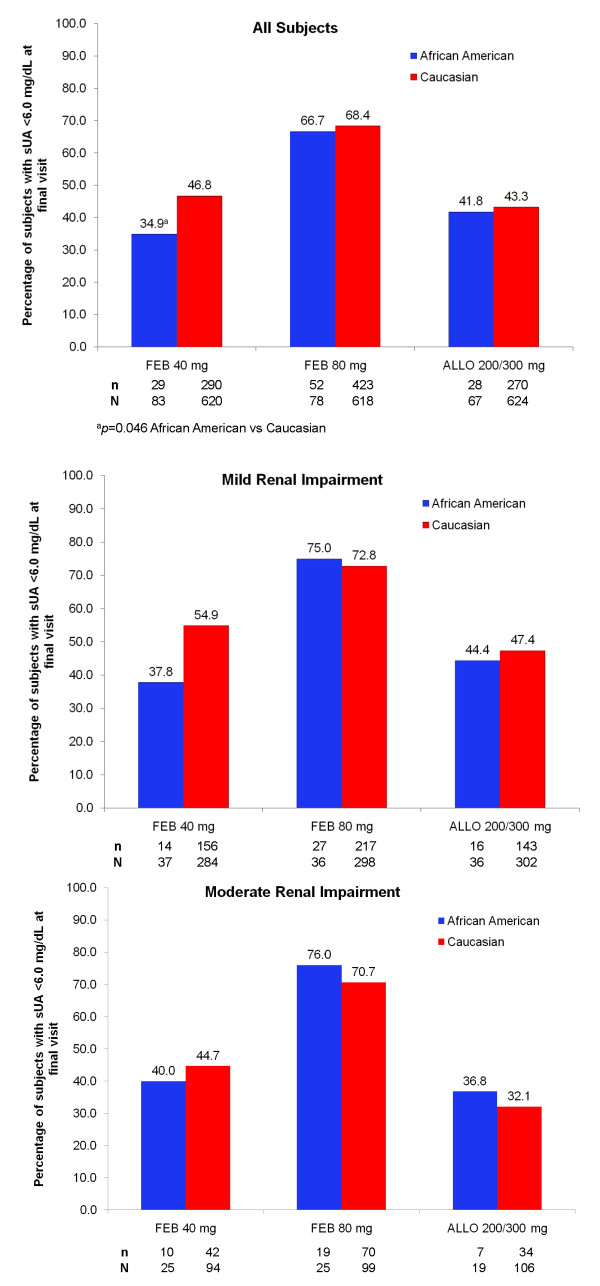
**Comparative Efficacy of Urate-Lowering Therapy Between African American and Caucasian Subjects Within Treatment Groups: A) All Subjects; B) Subjects With Mild Renal Impairment (eCLcr 60 to < 90 mL/min); and C) Subjects With Moderate Renal Impairment (eCLcr 30 to < 60 mL/min)**.

The primary efficacy endpoint of this analysis, sUA < 6.0 mg/dL at final visit, was achieved by 34.9%, 66.7%, and 41.8% of African American subjects in the febuxostat 40 mg, febuxostat 80 mg, and allopurinol 200/300 mg groups, respectively. Febuxostat 80 mg was significantly more efficacious than both febuxostat 40 mg (*p *< 0.001) and allopurinol 200/300 mg (*p *= 0.004). Similarly, among Caucasian subjects, significantly higher proportions of subjects in the febuxostat 80 mg group (68.4%) achieved sUA < 6.0 mg/dL compared to those in the febuxostat 40 mg group (46.8%; *p *< 0.001) and in the allopurinol 200/300 mg group (43.3%; *p *< 0.001). No statistical difference was observed in the urate-lowering efficacy rate between febuxostat 40 mg and allopurinol 200/300 mg in either the African American or Caucasian subgroup. Figure 2A provides comparisons of the achievement of the primary endpoint between African American and Caucasian subjects within each treatment group. Achievement of the primary efficacy endpoint was comparable between African American and Caucasian subjects when compared within treatment groups for either febuxostat 80 mg or allopurinol 200/300 mg. Although febuxostat was effective in African Americans, significantly less African American subjects achieved sUA < 6.0 mg/dL with febuxostat 40 mg than did Caucasian subjects (*p *= 0.046).

In both African American and Caucasian subjects with mild renal impairment, the urate-lowering efficacy of febuxostat 80 mg was greater than that of either febuxostat 40 mg (*p *= 0.002 in African Americans; *p *< 0.001 in Caucasians) or allopurinol 200/300 mg (*p *= 0.016 in African Americans; *p *< 0.001 in Caucasians). The same pattern was also observed in subjects with moderate renal impairment. Figures 2B/C provide comparisons in efficacy between African American and Caucasian subjects with mild or moderate renal impairment within each treatment group; efficacy rates between African American and Caucasian subjects within each treatment group were comparable.

In the febuxostat 40 mg, febuxostat 80 mg, and allopurinol 200/300 mg groups, 30%, 31%, and 30% of African Americans, respectively, and 30%, 31%, and 25% of Caucasians, respectively, required treatment for acute gout flares during the 6 months of the study.

Overall rates of AEs were comparable across treatment groups for both African American and Caucasian subjects. At least 1 AE was reported in the febuxostat 40 mg, febuxostat 80 mg, and allopurinol 200/300 mg groups by 45.8%, 60.3%, and 44.8% of African American subjects, respectively, and by 57.3%, 53.4%, and 58.7% of Caucasian subjects, respectively. Table 2 lists the most frequently reported AEs for African American and Caucasian subjects.

**Table 2:** Most Frequently Reported Adverse Events and Elevated Serum Liver Function Tests in African American and Caucasian Subjects

	African American N = 228	Caucasian N = 1863	*p *value^a^
	
Total subjects reporting ≥ 1 AE, *n (%)*	115 (50.4)	1052 (56.5)	0.090
**Most frequently reported AEs**, *n (%)*			
Liver function analyses	8 (3.5)	141 (7.6)	0.020
Upper respiratory tract infections	15 (6.6)	141 (7.6)	0.689
Diarrhea	10 (4.4)	128 (6.9)	0.202
Musculoskeletal and connective tissue signs and symptoms	9 (3.9)	89 (4.8)	0.739
Joint related signs and symptoms	7 (3.1)	69 (3.7)	0.851

**Elevated liver function tests**, *n/N (%)*			
ALT			
≥ 2X ULN	5/206 (2)	172/1737 (10)	< 0.001
≥ 3X ULN	1/206 (< 1)	50/1737 (3)	0.037
AST			
≥ 2X ULN	8/206 (4)	93/1736 (5)	0.505
≥ 3X ULN	3/206 (1)	24/1736 (1)	0.760

AE = adverse event; ALT = alanine aminotransferase; AST = aspartate aminotransferase; ULN = upper limit of normal

^a^*p *values are from Fisher's exact test

AEs are reported by MedDRA High Level Term. Most frequent events are those occurring in at least 3 percent of African American subjects.

Overall, rates of serious AEs were comparable across treatment groups in African American subjects as well as in the Caucasian subjects. Among African American subjects, 3.6%, 3.8%, and 4.5% in the febuxostat 40 mg, febuxostat 80 mg, and allopurinol 200/300 mg groups, respectively, reported at least 1 serious AE, while 2.3%, 3.9%, and 4.3% of Caucasian subjects, respectively, reported at least 1 serious AE. One (1.2%) African American subject, in the febuxostat 40 mg group, reported a cardiac serious AE. Among Caucasian subjects, cardiac serious AEs were reported by 3 (0.5%), 5 (0.8%), and 5 (0.8%) subjects in the febuxostat 40 mg, febuxostat 80 mg, and allopurinol 200/300 mg groups, respectively. Five subjects died during the CONFIRMS trial [23]--2 were African American (1 from sudden death and the other from necrotizing sepsis after surgery for lung carcinoma) and 3 were Caucasian (1 hypertensive heart disease, 1 anaphylactic shock from fire ant stings, and 1 brain edema and chronic pulmonary lung disease). No death was considered by investigators to be related to study drug.

## Discussion

Differences in the efficacy and safety of various drugs in different racial groups have been well documented and can be attributed to differing rates of comorbid conditions, concomitant medication use, and underlying genetic variations in the enzymes involved in drug metabolism [27]. For example, dosing adjustments for warfarin are recommended in African American patients due to decreased metabolism of the drug, which can lead to increased risk for bleeding. Clinical and genetic components that may affect warfarin metabolism in African Americans include age, weight, cerebrovascular disease, and the presence of certain variants of the hepatic isoenzyme cytochrome P450 (CYP) 2C9, the primary metabolizer of warfarin [28]. Febuxostat is extensively metabolized in the liver by conjugation via uridine diphosphate glucuronosyltransferase (UGT) enzymes, including UGT1A1, UGT1A3, UGT1A9, and UGT2B7, and, to a much lesser extent, oxidation via CYP1A2, 2C8, 2C9, and non-P450 enzymes [20]. Different gene variants for UGT1A1, UGT1A9, and CYP2C9 have been identified in African Americans vs Caucasians [29-31]. However, based on our results in this limited number of African American subjects, these genetic variants do not appear to affect the metabolism of febuxostat in such a way that would interfere with its efficacy.

The higher rates of gout noted in the African American population [5,6] have been attributed to high rates of comorbid conditions associated with increased risk for gout [10], such as diabetes, hypertension, obesity, and renal disease [8]. Along with clinical differences that may contribute to increased risk for developing gout, identification of underlying genetic differences in the various enzymes and transporters involved in purine metabolism and urate renal excretion could shed further light on why African Americans are affected with gout at higher rates. For example, a number of renal urate transporters and their genes have been identified. Variants of these genes influence sUA. While some of these genetic variants strongly influence sUA in both Caucasians and African Americans, others are more specifically associated with one race or the other [32-34].

There is a growing body of evidence that both hyperuricemia and gout increase the risk for the development and/or progression of renal dysfunction, cardiovascular disease, hypertension, metabolic syndrome, and diabetes [35-40], and all-cause and cardiovascular-related mortality [41,42]. In addition, the evidence suggests a disparity between African Americans and Caucasians. In the Atherosclerosis Risk in Communities Study, a prospective epidemiological cohort study, increasing sUA as a continuous variable--after adjusting for age, baseline blood pressure, BMI, renal function, diabetes, and smoking--was shown to significantly increase the risk for the development of hypertension in African Americans, but not for Caucasians, regardless of concomitant medication use [43]. In another such study, each unit increase in sUA was associated with a higher risk for cardiovascular mortality in African American men and women compared to their Caucasian counterparts [44].

Proper management of the underlying hyperuricemia of gout is necessary for the proven reduction in the clinical manifestations of the disease, including gout flares and tophi [14,15,45-47]. Although not approved for such use, treatment with allopurinol or febuxostat has also been shown to ameliorate renal damage induced by hyperuricemia in rats [48,49], and to stabilize or even improve renal function in humans [50-53]. A recent study in humans has also demonstrated the cardiovascular-protective impact of lowering sUA levels [35]. Therefore, proper management of African American gout patients goes beyond the acute treatment of flares, tophi, or kidney stones and incorporates effective reduction and maintenance of sUA to target levels of < 6.0 mg/dL.

While the clinical benefits of reducing sUA long-term likely extend beyond relief from gout, providing optimum management to African American gout patients may be challenging. Data from the National Ambulatory Medical Care Survey reveal that of 3.9 million outpatient visits with a gout diagnosis that occurred in the US during 2002, only 10% were made by African Americans vs 82% by Caucasians [7]. Caucasians with a gout visit were more likely to have private insurance (46%) compared to African Americans (11%; p < 0.001) and, importantly, African Americans were less likely than Caucasians to receive ULT with allopurinol (odds ratio [OR] 0.18; 95% CI, 0.04-0.78; *p *= 0.02) [7]. In addition, African American patients with gout are more likely to be non-adherent with ULT than Caucasian patients (OR 1.86; 95% CI, 1.52-2.27) [54]. Interestingly, we observed in this analysis that the African American subjects were 3-times more likely to be lost to follow up than the Caucasian subjects (9.6% vs 3.0%, respectively) and less adherent with therapy (72.4% vs 82.1%, respectively).

There are no published studies specifically examining racial disparities in the diagnosis and management of gout. Therefore possible explanations for low rates of gout diagnosis despite higher risk, lower use of ULT, and lower compliance with therapy among African Americans compared to Caucasians can only be inferred from other chronic diseases. In a 2002 report on ethnic disparities in arthritis and musculoskeletal diseases [55], Jordan et al. attributed some disparities to ethnic differences in access to care, care-seeking behavior, and utilization of care. According the 2010 National Healthcare Disparities Report [56], healthcare quality and access continue to be suboptimal for minority and low-income groups. Perceived provider discrimination, which is higher among minorities, can lead to delay in seeking health care [57]. There are noted racial differences in treatment preferences for rheumatoid arthritis; African American patients attach greater importance to the risks of toxicity and less importance to the likelihood of benefit than their Caucasian counterparts [58,59]. Similarly, among patients with at least moderately severe osteoarthritis, African Americans were significantly less likely than Caucasians to perceive the benefit of total joint arthroplasty and more likely to recognize barriers to the procedure [60]. Based on just the above small sampling of the literature, it is likely that the underlying reasons for racial disparities in gout are multifactorial and require investigation.

ULT with febuxostat 80 mg was significantly better than either febuxostat 40 mg or allopurinol 200/300 mg in the African American cohort of hyperuricemic gout subjects with high rates of comorbidities. This was also observed in the Caucasian cohort and reflects the overall results of the CONFIRMS trial [23]. Similarly, among both African Americans and Caucasians with mild or moderate renal impairment, febuxostat 80 mg was significantly better at achieving sUA < 6.0 mg/dL compared to either febuxostat 40 mg or allopurinol 200/300 mg.

When the efficacy of each treatment group was compared between African American and Caucasian subjects, the only significant difference observed was in the febuxostat 40 mg treatment group, with lower efficacy observed in African American subjects in the overall cohort (34.9% vs 46.8%; *p *= 0.046). One plausible explanation for this observed difference may be the noted difference in compliance with treatment. Within the febuxostat 40 mg group, Caucasian subjects had a considerably higher compliance rate (82.4%) than their African American counterparts (66.3%). This difference was greater than those observed in the other 2 treatment groups. In addition, a large numerical difference was observed in subjects with mild renal impairment (37.8% vs 54.9%) but this did not reach statistical significance (*p *= 0.055). The lack of significant difference is likely due to the small number of African American subjects. In addition, no significant differences were observed between African American and Caucasian subjects with mild or with moderate renal impairment in the efficacy of febuxostat 80 mg or allopurinol 200/300 mg. In each treatment group the percentages of African American and Caucasian subjects that required treatment for gout flares were comparable. Flare rates during initial ULT correlate with the extent of sUA decrease [61], therefore similar rates reflect comparable efficacy between the two groups. Along with comparable efficacy, ULT with either dose of febuxostat or allopurinol 200/300 mg was well-tolerated by both African Americans and Caucasian subjects.

Limitations of this subanalysis include its post-hoc nature and the low number of African Americans enrolled in the CONFIRMS trial compared to Caucasians. Despite the fact that they develop gout at higher rates than Caucasians [5,6], in the CONFIRMS trial, along with other ULT RCTs, African Americans comprised ≤ 10% of the study population [21-23]. Underrepresentation of African Americans in the febuxostat clinical trials is not unique to the enrollment patterns observed for the majority of Phase 3 clinical intervention trials conducted in the US and reflects the continued hurdles faced by trial investigators in recruiting minority populations [62]. However, the data reported here represents the first report of ULT efficacy and safety in African American gout patients. Additional studies incorporating greater numbers of minorities are needed to confirm our results.

## Conclusions

ULT is equally efficacious in African American and Caucasian gout patients. For African American gout patients with mild or moderate renal impairment, febuxostat 80 mg is significantly more effective at lowering sUA to < 6.0 mg/dL than febuxostat 40 mg or the commonly prescribed doses of allopurinol (200 or 300 mg) [17].

## Competing interests

Alvin F. Wells has served as a consultant for Takeda Global Research & Development Center, Inc, Abbott Laboratories, Amgen, Inc, Bristol-Myers Squibb Company, Contactor, Inc, Eli Lilly and Company, GlaxoSmithKline, Pfizer, Inc, Wyeth Pharmaceuticals, and Genetech, Inc. Patricia A. MacDonald, Solomon Chefo, and Robert L. Jackson are all employees of Takeda Global Research & Development Center, Inc, and were employees of TAP Pharmaceutical Products, Inc, at the time of the study conduct.

## Authors' contributions

Alvin F. Wells was involved in the acquisition of data, data analysis and interpretation, and preparation of the manuscript. Patricia A. MacDonald, Solomon Chefo, and Robert L. Jackson were involved in study concept and design, acquisition of data, data analysis and interpretation, and preparation of the manuscript. All authors provided final approval to submit the manuscript. The study sponsor, Takeda Global Research & Development Center, Inc, was involved in the study design, protocols, subject recruitment, data collections, and analyses.

## Pre-publication history

The pre-publication history for this paper can be accessed here:

http://www.biomedcentral.com/1471-2474/13/15/prepub
